# Term breech presentation vaginal births in Tibet: A retrospective analysis of 451 cases

**DOI:** 10.3389/fmed.2023.1048628

**Published:** 2023-04-17

**Authors:** Fang Long, Keqing Yan, Dongxing Guo, Duoji Zhaxi, Xiaoguang Xu, Zhigang Sun, Zhen Xiao

**Affiliations:** ^1^Department of Obstetrics and Gynecology, First Affiliated Hospital of Dalian Medical University, Dalian, China; ^2^Department of Obstetrics and Gynecology, People’s Hospital of Naqu, Tibet, China; ^3^Research Center of High Altitude Medicine of Naqu, Tibet, China; ^4^Institute of High Altitude Medicine, People’s Hospital of Naqu, Tibet, China; ^5^Department of Pathology, First Affiliated Hospital of Dalian Medical University, Dalian, China

**Keywords:** term breech birth, Tibet, neonatal mortality, perineal laceration, stages of labor

## Abstract

**Background:**

In high altitude areas, like Tibet, most fetuses in breech presentation at term are delivered vaginally owing to a variety of reasons, but this has not been published.

**Objective:**

This study aimed to provide references and evidence for the delivery of breach presentation term fetuses in high altitude areas, through comparing and analyzing the data of full-term singleton fetuses with breech or cephalic presentation in Naqu People’s Hospital, Tibet.

**Study design:**

We retrospectively analyzed the clinical data of 451 breech presentation fetuses mentioned above over a period of 5  years (2016–2020). A total of 526 cephalic presentation fetuses’ data within 3  months (1 June to 1 September 2020) of the same period were collected too. Statistics were compared and assembled on fetal mortality, Apgar scores, and severe neonatal complications for both planned cesarean section (CS) and vaginal delivery. In addition, we also analyzed the types of breech presentation, the second stage of labor, and damage to the maternal perineum during vaginal delivery.

**Results:**

Among the 451 cases of breech presentation fetuses, 22 cases (4.9%) elected for CS and 429 cases (95.1%) elected for vaginal delivery. Of the women who chose vaginal trial labor, 17 cases underwent emergency CSs. The perinatal and neonatal mortality rate was 4.2% in the planned vaginal delivery group and the incidence of severe neonatal complications was 11.7% in the transvaginal group, no deaths were detected in the CS group. Among the 526 cephalic control groups with planned vaginal delivery, the perinatal and neonatal mortality was 1.5% (*p* = 0.012), and the incidence of severe neonatal complications was 1.9%. Among vaginal breech deliveries, most of them were complete breech presentation (61.17%). Among the 364 cases, the proportion of intact perinea was 45.1%, and first degree lacerations accounted for 40.7%.

**Conclusion:**

In the Tibetan Plateau region, vaginal delivery was less safe than cephalic presentation fetuses for full-term breech presentation fetuses delivered in the lithotomy position. However, if dystocia or fetal distress can be identified in time and then encouraged to convert to cesarean, its safety will be greatly improved.

## 1. Introduction

Since the 1970s, the CS rate of fetal breech presentation has risen rapidly (1), in California, United States it rose from 12% in 1970 to 95% in 1999 (2–5). After the Term Breech Trial (6), in some countries including the Netherlands, Sweden, Finland, Norway, and France, a marked increase in the CS rate for term singleton breech presentation fetuses was observed (1, 7). Currently, most of the breech presentations in China undergo CS, with the rate in a hospital in Beijing being as high as 90.68% (8).

However, in Tibet, most breech presentations are still delivered vaginally. The main reasons are as follows: (1) Most of the pregnant women are multipara, and have a high number of births. The progression of their labor is relatively fast as, following the long distance they have usually travelled, by the time they arrive at the hospital the cervical opening is often widely opened; (2) Influenced by traditional beliefs, many pregnant women refuse to have a CS; (3) Some women are concerned that CS may adversely affect a subsequent pregnancy; (4) Tibet is located at a high altitude with consequent lower oxygen levels on the plateau; therefore, fetal weights are lower than in the plain area (an average of 96.98 g lower birth weight for every 1,000 m in elevated maternal altitude) (9, 10), which can make it relatively easier to deliver breech presentations vaginally; (5) Some regions are relatively short of the medical resources needed for CS. Because of the special situation in Tibet, the analysis of our data can reflect the natural state of breech presentation vaginal delivery without intervention. This study aimed to provide a reference and basis for the delivery of full-term breech presentation fetuses in high altitude areas.

In recent years, the neonatal mortality rate in Tibet has dropped significantly, but it still ranks highest in China. A study has shown that the leading causes of neonatal mortality in Tibetan plateau areas include perinatal disease and asphyxia during childbirth (11). Vaginal delivery during breech presentation is undoubtedly one of the risk factors for neonatal complications and death. Therefore, this study creates a guideline for analyzing the causes of neonatal death and reducing the overall mortality rate in Tibet.

## 2. Materials and methods

From 2016 to 2020, a total of 666 breech presentation fetuses were delivered in our hospital. Excluding non-term fetuses and intrauterine stillbirths, a total of 451 singleton breech presentation fetuses were studied. Of them, 429 had elected a vaginal delivery. The exclusion criteria of breech vaginal delivery were as follows: (i) The umbilical cord was located under the fetal presentation; (ii) fetal growth was restricted (we chose the lowest neonatal birth weight of 2,500 g as the FGR standard); (iii) Suspicious macrosomia (ultrasound estimated fetal weight ≥3,800 g); (iv) Fetal presentation size disproportionate to maternal pelvic size; (v) fetal malformations that obstructed vaginal delivery; (vi) the fetal head was overextended; (vii) the woman refused vaginal trial labor (12, 13). The remaining 22 cases elected a planned CS.

We compared neonatal outcomes of planned CS with planned vaginal delivery, and also compared it with emergency CS following failed vaginal trial labor.

At the same time, a total of 526 cases’ clinical data of full-term cephalic fetuses with planned vaginal delivery delivered in Naqu People’s Hospital (June 1 and September 1, 2020) were collected as cephalic control group. All cases had elected a vaginal delivery. Neonatal outcomes for cephalic versus breech presentation delivery were analyzed.

The study design is shown in Figure 1.

**Figure 2:**
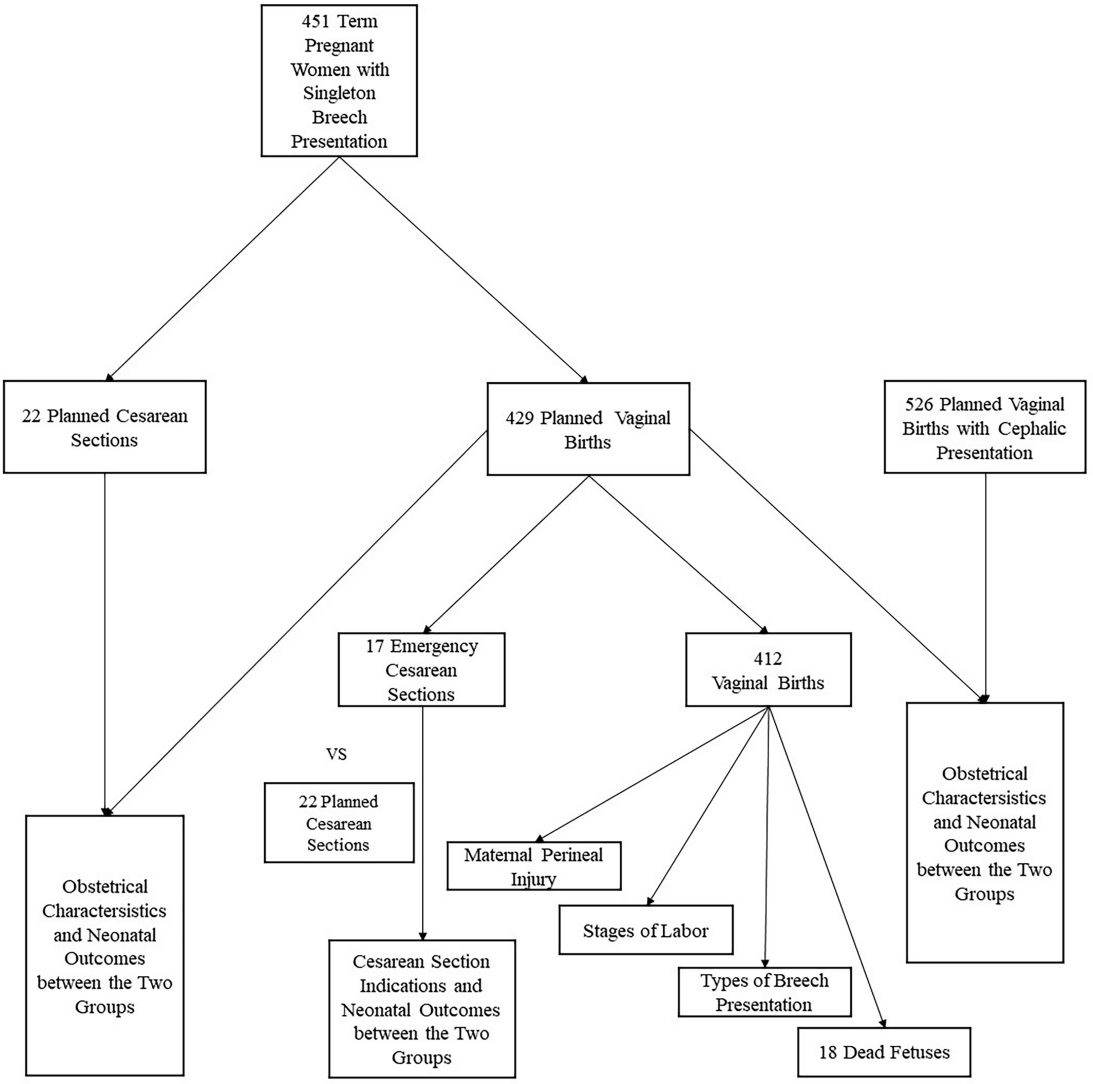
Comparison between two modes of breech delivery. **(A–C)** Shows a comparison of body weight of newborns, maternal age and gestational age between the planned vaginal delivery group and the planned cesarean delivery group (*p* = 0.215, 0.610, 0.478, respectively). **(D)** Shows the number of multiparous and nulliparous between two modes of delivery (*p* = 0.056).

For each case, experienced obstetricians and pediatricians were present during the vaginal deliveries. The breach fetuses were delivered spontaneously as far as the umbilicus, and the remainder of the body was extracted or delivered with obstetrician’s traction and assisted maneuvers, including the Bracht method (14), the Mauriceau Maneuver (15) and etc. All women who delivered vaginally used the classic lithotomy position. No anesthesia was used during labor in the vaginal delivery group. We assessed labor progress by vaginal examination: every 2–4 h in the first stage of labor and every 2 h in the second stage. More frequent examinations could be performed when there was a concern about labor progress (16). Oxytocin was used only when the woman was considered to have secondary atony leading to prolonged labor or fetal distress. The dose of oxytocin was 2.5 U, which was added to 500 ml of glucose and administered by intravenous infusion. Moreover, during vaginal trial labor, the fetal heart rate was monitored throughout using an electronic fetal heart rate monitor. When fetal heart rate monitoring indicated intrauterine hypoxia, the mother was asked to inhale oxygen, she was left in the decubitus position, and was given supplemental nutrition to correct the hypoxia state.

All cephalic vaginal delivery positions also used the classic lithotomy position. Assessment and management of labor were the same as for breech delivery.

The main measures for assessing neonatal outcomes were: perinatal and neonatal mortality (death during childbirth and within 7 days), Apgar score (1 min and 5 min) and severe neonatal morbidity (neonatal encephalopathy, respiratory distress syndrome, intracranial hemorrhage, pneumothorax, joint dislocation, omphalitis, and hyperbilirubinemia).

Neonatal encephalopathy (NE) is a clinically defined syndrome of disturbed neurologic function in the earliest days of life in an infant born at or beyond 35 weeks of gestation, manifested by a subnormal level of consciousness or seizures, and often accompanied by difficulty with initiating and maintaining respiration and depression of tone and reflexes (17). Our diagnosis of NE was mainly based on neuro-psychiatric symptoms and auxiliary examinations such as electroencephalogram (EEG) and MRI.

In addition, we analyzed the duration of the second stage of labor and maternal perineal injuries of breech presentation fetuses by vaginal delivery. The second stage of labor refers to the interval between full cervical dilatation (10 cm) and delivery of the infant; a primipara needed about 3 h and the multipara did not exceed 2 h (18, 19). The classification of perineal lacerations was as follows: (i): Injury to perineal skin only; (ii): injury to the perineum involving perineal muscles but not involving the anal sphincter; (iii): injury to the perineum involving the anal sphincter complex; (iv): injury to the perineum involving the anal sphincter complex (external anal sphincter and internal anal sphincter) and anal epithelium (20).

Different types of breech presentation are described: frank breech and incomplete or complete breech. In a frank breech, the fetus has flexion of both hips, and the legs are straight with the feet near the fetal face. The complete breech has the fetus sitting with flexion of both hips and both legs in a tuck position. Finally, the incomplete breech can have any combination of one or both hips extended (21).

The data were analyzed in this study based on the mode of final delivery. Comparisons were carried out by chi-square test or Fisher’s precision test, and the comparison of numbers was *t*-tested, using *p* < 0.05 to denote statistical significance. Data analysis was performed using SPSS software (26.0).

## 3. Results

**Figure 3:**
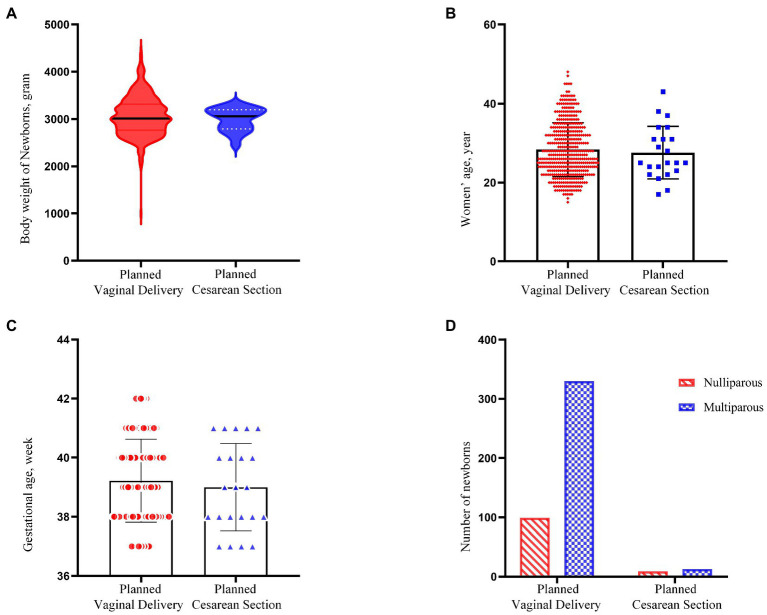
Comparison between breech and cephalic presentation. **(A–C)** Shows a comparison of body weight of newborns, maternal age and gestational age between the breech group and cephalic group (*p* = 0.115, 0.761, 0.052, respectively). **(D)** Shows the number of multiparous and nulliparous between two groups (*p* = 0.868).

**Figure 4:**
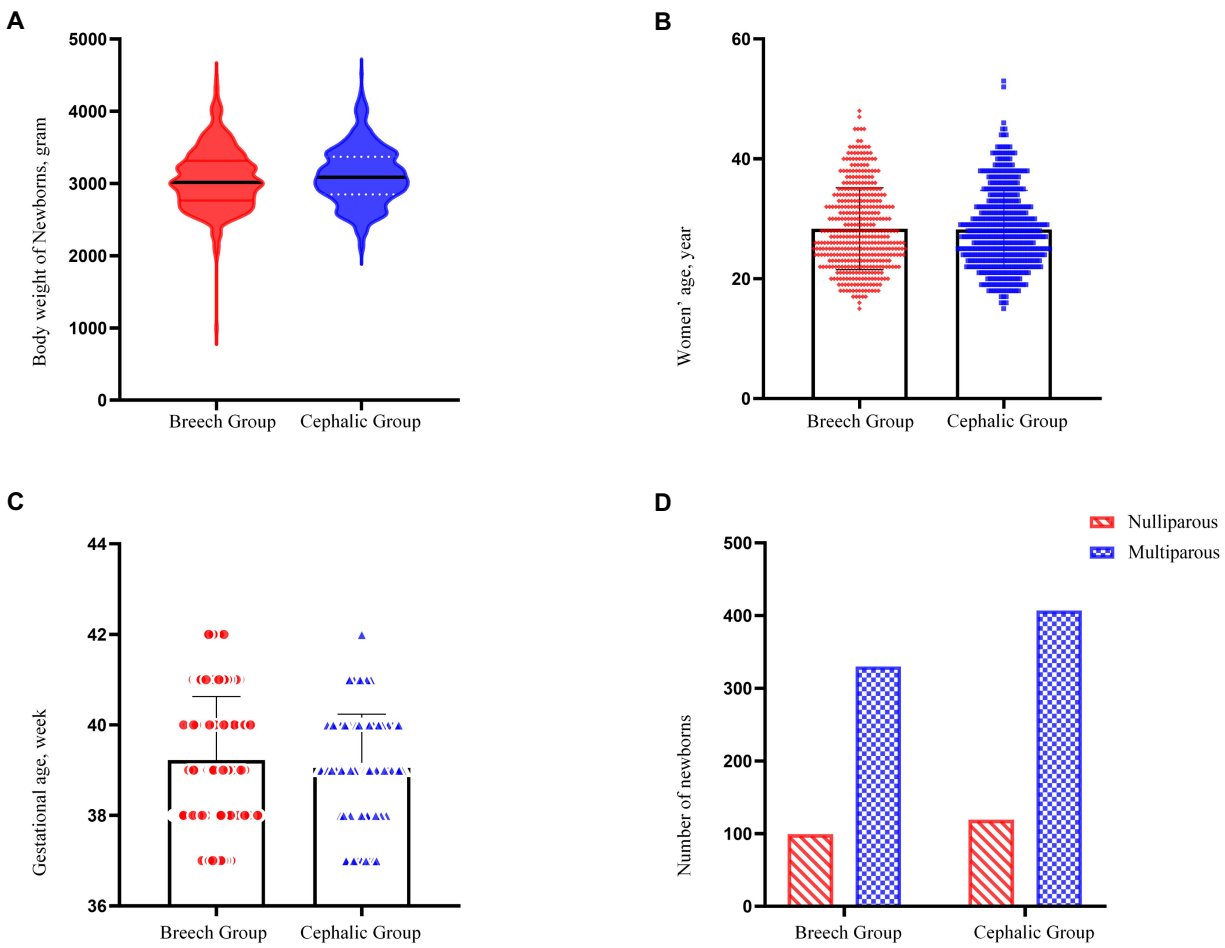
Cesarean section indication. All planned cesarean sections were indicated for breech presentation and refusing to undergo vaginal trial labor. Premature rupture of membranes accounts for the largest proportion of emergency caesarean sections. **A** and **B** indicate surgical indications for planned cesarean section versus emergency cesarean section, respectively

The basic features and neonatal outcomes between planned vaginal delivery versus planned CS of breech presentation fetuses, and between cephalic and breech presentation fetuses with planned vaginal delivery, were analyzed. The basic characteristics of the two comparison groups are shown in Tables 1, 2 and Figures 2, 3. Over the last 5 years, 451 cases of term singleton breech fetuses were delivered in our hospital; among these, 429 (95.1%) had elected for vaginal delivery and 22 (4.9%) had elected for CS. Planned CSs are indicated where the presentation is breech or where the patient refused a vaginal trial. Of women in the elected vaginal delivery group, 17 eventually had an emergency CS due to unsuccessful delivery. The predominant indications for CS were premature rupture of membranes (58.8%), followed by pregnancy-induced hypertension (17.6%), scarred uterus (17.6%), and acute intrauterine fetal distress (5.9%), as shown in Figure 4. Of the 526 cephalic births collected, 24 were converted to emergency CS (4.6%), similar to breech births (4.0%).

**Table 1:** Characteristics of term breech delivery in the study.

Characteristic	Planned vaginal delivery (*n* = 429, 95.1%)	Planned cesarean delivery (*n* = 22, 4.9%)	95% CI or *X*^2^		*p* value
Maternal age (years)	28.4 ± 6.8	27.6 ± 6.7	−2.165	3.687	0.610
Nulliparous (No. %)	99 (23.1%)	9 (40.9%)	3.650		0.056
Multiparous (No. %)	330 (76.9%)	13 (59.1%)			
Gestational age (weeks)	39.2 ± 1.41	39.0 ± 1.48	−0.387	0.825	0.478
Birth weight (g)	3,066 ± 418	2,995 ± 243	−43.388	184.434	0.215
Birth weight ≥3,800 g (No.)	20 (4.7%)	0 (0.0%)	Fisher		0.614
Birth weight <2,500 g (No.)	17 (4.0%)	1 (4.5%)	Fisher		0.601

**Table 2:** Characteristics between term breech and cephalic delivery in the study.

Characteristic	Breech presentation (*n* = 429)	Cephalic presentation (*n* = 526)	95% CI or *X*^2^		*p* value
Maternal age (years)	28.4 ± 6.8	28.2 ± 6.5	−0.718	0.980	0.761
Nulliparous (No. %)	99 (23.1%)	119 (22.6%)	0.028		0.868
Multiparous (No. %)	330 (76.9%)	407 (77.4%)			
Gestational age (weeks)	39.2 ± 1.4	39.1 ± 1.2	−0.002	0.333	0.052
Birth weight (g)	3,066 ± 418	3,107 ± 397	−93.551	10.205	0.115
Birth weight ≥3,800 g (No.)	20 (4.7%)	24 (4.6%)	0.005		0.942
Birth weight <2,500 g (No.)	17(4.0%)	20(3.8%)	0.016		0.898

**Figure 5:**
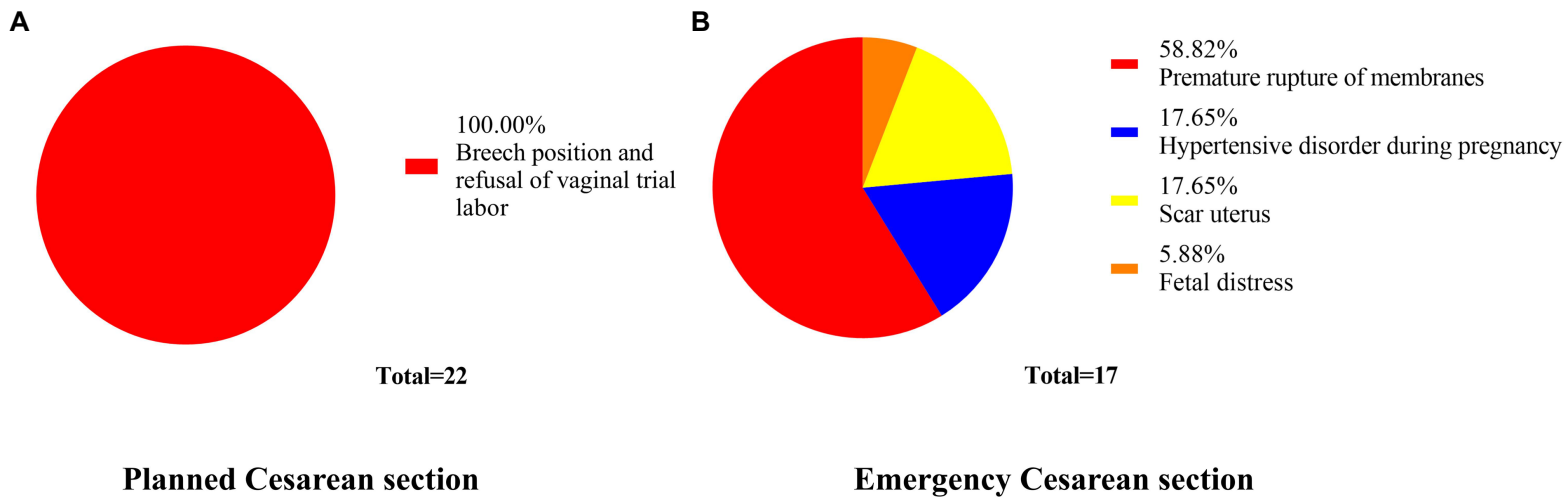
Apgar score between two modes of breech delivery. This compared the one-minute and five-minute Apgar score in the planned vaginal delivery group with that in the planned cesarean section group. The number of Apgar scores <7 at 1 min in vaginal delivery infants was significantly higher than that in cesarean section group (*p* < 0.01). The same difference between the two groups was not statistically significant at 5 min (*p* = 0.096). In the cesarean section group, there were no infants with Apgar score <7 in 5 min. **A** represents the difference in 1-minute Apgar score between the cesarean section group and the vaginal delivery group; and **B** represents the 5-minute Apgar score difference between the two groups.

Comparisons of the main neonatal outcomes are shown in Tables 3–5. The number of low Apgar scores at 1 min in vaginal delivery infants was significantly higher than that in the CS group, and the difference was statistically significant (*p* < 0.01). However, the difference between the two groups was not statistically significant at 5 min (Figure 5). In the cesarean group, there were no deaths or infants with Apgar score <7 at 5 min. Most stillbirths or neonatal deaths were caused by fetal hypoxia during labor, and intrapartum deaths accounted for 61.1% of all deaths. The specifics of the 18 dead fetuses are shown in Table 6. There were no serious neonatal complications in the elected CS group, while the incidence of serious neonatal complications in the elected vaginal delivery group was 4.2%; however, there was no statistical significance between the two groups. In the group planning for vaginal delivery who were transferred to neonatal treatment, 42.3% of them were cured or discharged from hospital; whereas 40.4% of infants requiring treatment were abandoned by their parents because of lack of money.

**Table 3:** Primary outcome of term infants in breech presentation between planned vaginal delivery and cesarean delivery.

	Planned vaginal delivery (*n* = 429, 95.1%)	Planned cesarean delivery (*n* = 22, 4.9%)	*X* ^2^	*p* value
Perinatal or neonatal death	18 (4.2%)	0	Fisher	1.000
Sever neonatal morbidity	50 (11.7%)	0	1.823	0.177
Neonatal encephalopathy	46 (10.7%)	0	1.590	0.208
Intracranial hemorrhage	16 (3.7%)	0	Fisher	1.000
Dislocation of joint	1 (0.2%)	0	Fisher	1.000
Pneumothorax	1 (0.2%)	0	Fisher	1.000
Respiratory distress syndrome	3 (0.7%)	0	Fisher	1.000
Omphalitis	1 (0.2%)	0	Fisher	1.000
Hyperbilirubinemia	5 (1.2%)	0	Fisher	1.000
**Apgar score**				
<7 at 1 min	194 (45.6%)	1 (4.5%)	14.368	***p* < 0.01**
<4 at 1 min	151 (35.5%)	0	11.804	***p* < 0.01**
<7 at 5 min	65 (15.3%)	0	2.803	0.094
<4 at 5 min	17 (4.0%)	0	Fisher	1.000
Admission to the neonatal care unit	49 (11.4%)	0	1.763	0.184
**Pediatric treatment**		–	–	–
Healing	22 (42.3%)	0	–	–
Transfer	3 (5.8%)	0	–	–
Death	6 (11.5%)	0	–	–
Give up treatment	21 (40.4%)	0	–	–

“*p* <0.01” is used to highlight that the value is statistically significant.

**Table 4:** Primary outcome of term infants between breech and cephalic presentation.

	Breech presentation (*n* = 429)	Cephalic presentation (*n* = 526)	*X* ^2^	*p* value
Convert to cesarean delivery	17 (4.0%)	24 (4.6%)	0.207	0.649
Perinatal or neonatal death	18 (4.2%)	8 (1.5%)	6.384	**0.012**
Sever neonatal morbidity	50 (11.7%)	10 (1.9%)	38.179	***p* < 0.01**
Neonatal encephalopathy	46 (10.7%)	8 (1.5%)	37.503	***p* < 0.01**
Intracranial hemorrhage	16 (3.7%)	1 (0.2%)	16.931	***p* < 0.01**
Dislocation of joint	1 (0.2%)	0	Fisher	0.449
Pneumothorax	1 (0.2%)	0	Fisher	0.449
Respiratory distress syndrome	3 (0.7%)	1 (0.2%)	0.502	0.479
Omphalitis	1 (0.2%)	0	Fisher	0.449
Hyperbilirubinemia	5 (1.2%)	1 (0.2%)	2.208	0.137
**Apgar score**				
<7 at 1 min	194 (45.6%)	24 (4.6%)	224.568	***p* < 0.01**
<4 at 1 min	151 (35.5%)	13 (2.5%)	180.006	***p* < 0.01**
<7 at 5 min	65 (15.3%)	12 (2.3%)	53.492	***p* < 0.01**
<4 at 5 min	17 (4.0%)	4 (0.8%)	11.424	***p* < 0.01**

“*p* <0.01” is used to highlight that the value is statistically significant.

**Table 5:** Primary outcome of term infants in breech presentation between emergency and planned cesarean.

	Emergency cesarean delivery (*n* = 17)	Planned cesarean delivery (*n* = 22)	*X* ^2^	*p* value
Sever neonatal morbidity	1 (5.9%)	0	Fisher	0.436
Neonatal encephalopathy	1 (5.9%)	0	Fisher	0.436
**Apgar score**				
<7 at 1 min	4 (23.5%)	0	3.495	0.062
<4 at 1 min	1 (5.9%)	0	Fisher	0.436

**Figure 6:**
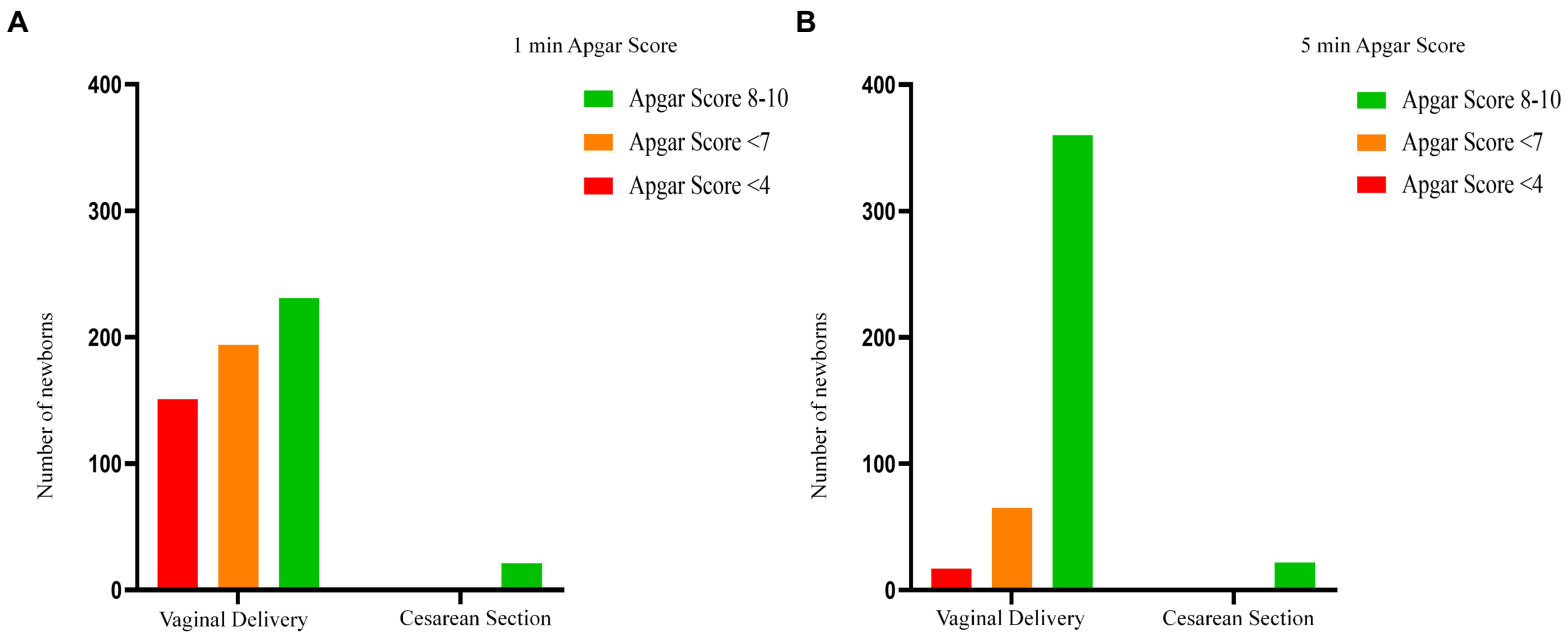
Apgar score between breech and cephalic presentation. This compared the one-minute and five-minute Apgar score in the breech group with that in the cephalic group. The number of Apgar scores <7 at 1 min and 5 min in breech group was significantly higher than that in cephalic group (*p* < 0.01, *p* < 0.01, respectively). And the number of Apgar scores <4 at 1 min and 5 min in breech group was also significantly higher (*p* < 0.01, *p* < 0.01, respectively).

**Table 6:** Basic information of 18 dead fetuses.

Number	Women’s age (year)	Times of birth	Gestational week	Breech presentation	First stage of labor (h)	Second stage of labor (min)	Maternal perineal injury	Birth weight (g)	1 min Apgar score	5 min Apgar score	Transfer to pediatrics	Complication
1	28	4	40	Frank	10	60	I° PL^*^	4,450	1	4	Yes	NE^*^, IH^*^
2	28	3	40	Complete	5	10	I° PL	3,500	1	4	Yes	NE
3	19	3	42	Complete	10.5	80	No injury	3,500	1	3	Yes	AP^*^, ARDS^*^
4	45	2	37	Complete	1.5	30	I° PL	2,500	1	2	No	NE
5	20	1	38	Frank	7	20	I° PL	2,900	1	1	No	NE
6	26	3	38	Complete	6	10	No injury	2,900	10	10	Yes	AP^*^, ARDS^*^
7	23	1	38	Frank	7	70	PT^*^	2,600	1	1	No	NE
8	31	6	40	Complete	7	60	No injury	3,510	1	0	No	AX^*^
9	21	1	40	Frank	5	40	PT	3,500	1	0	No	AX
10	40	3	40	Complete	5	30	PT	3,300	1	0	No	AX
11	32	4	39	Complete	6.83	20	I° PL	3,010	1	0	No	AX
12	18	1	40	Frank	8	80	PT	3,000	1	0	No	AX
13	22	3	38	Complete	8	19	No injury	2,640	1	0	No	AX
14	41	4	37	Complete	6	35	I° PL	2,630	1	0	No	AX
15	41	5	41	Complete	6.5	60	PT	3,500			No	
16	26	2	39	Complete	7	30	No injury	3,000			No	
17	40	4	40	Frank	5	55	No injury	3,000			No	
18	17	1	39	Complete	8	60	III° PL	2,600			No	

^*^PL is the short form of perineal laceration; PT is the short form of perineotomy; NE is the short form of neonatal encephalopathy; IH is the short form of intracranial hemorrhage; AP is the short form of aspiration pneumonia; ARDS is the short form of acute respiratory distress syndrome; AX is the short form of asphyxia.

There was no significant difference in short-term neonatal outcomes between converted CS and elective CS in breech presentation group.

Both the perinatal mortality and severe neonatal morbidity was significantly higher in the breech group than in the cephalic group, and the incidence of neonatal encephalopathy and intracranial hemorrhage was statistically significant. There was a significant difference in the low Apgar score rate between the two groups (breech group was higher than cephalic group), which was statistically significant at either 1 min or 5 min (Figure 6).

**Figure 7:**
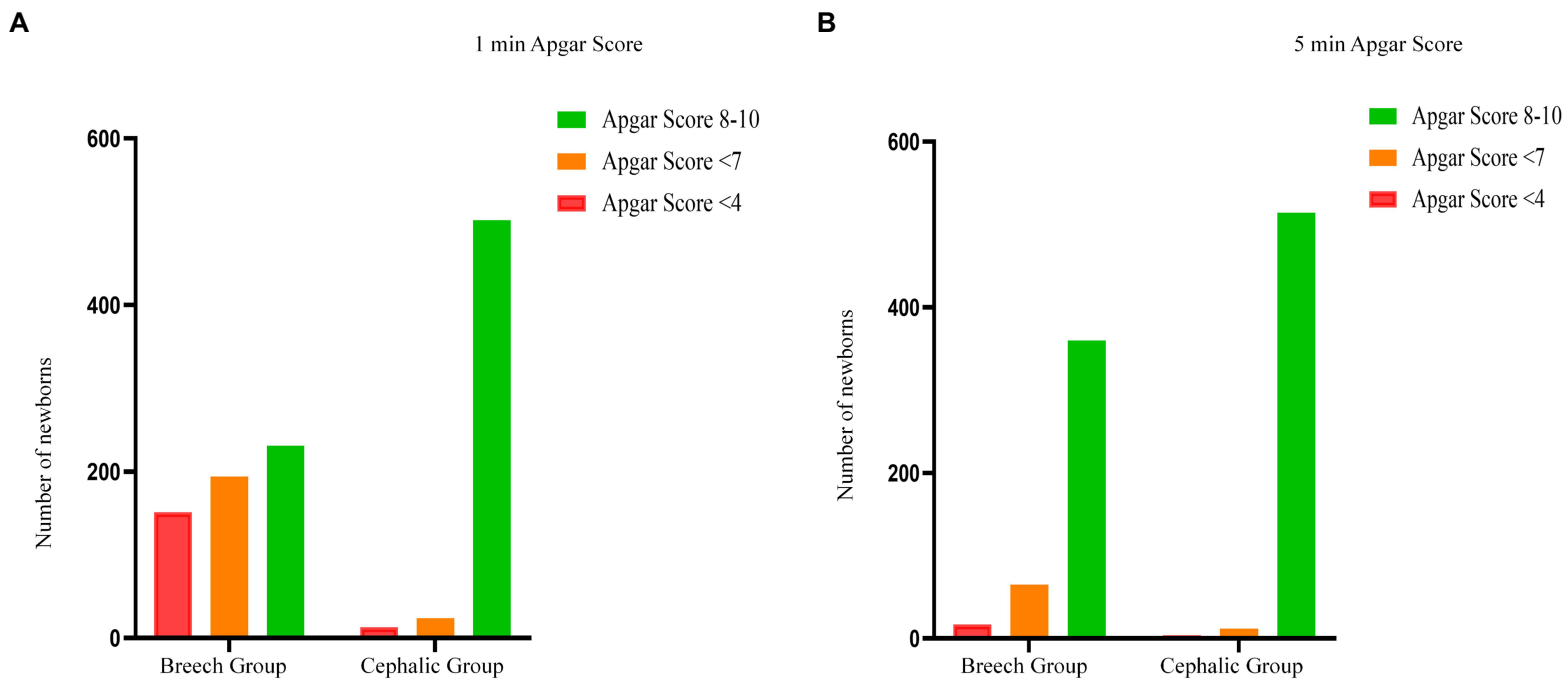
Types of breech presentation. This figure shows the type and proportion of the breech position of fetus in the vaginal delivery group. Most of these are complete breech presentation (*n* = 252, 61.17%).

**Figure 8:**
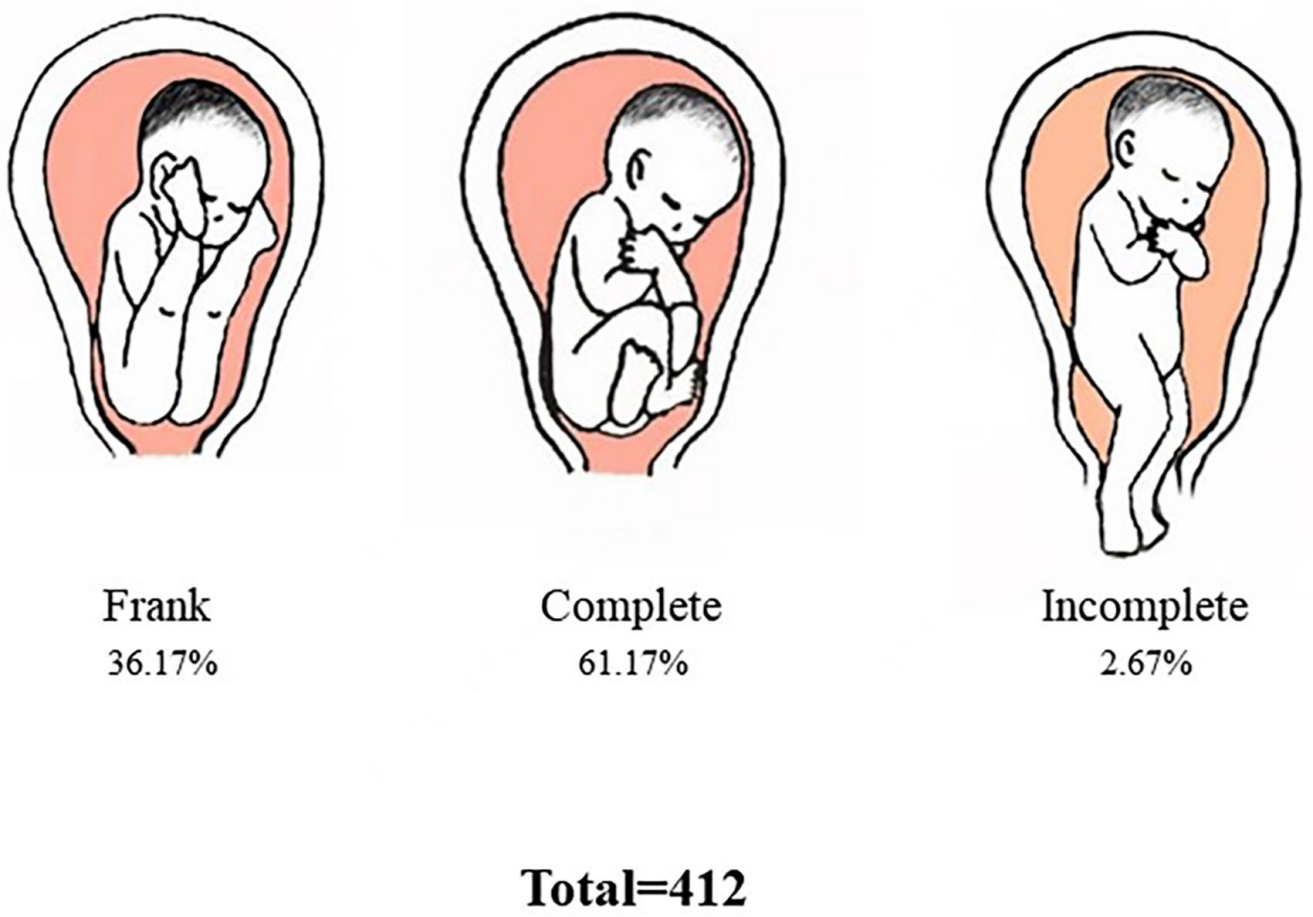
Maternal perineal injury. This shows perineal damage during vaginal delivery. Among the 364 women, most women had no perineal injury or had only first degree perineal laceration (45.1, 40.7%, respectively), and severe perineal laceration occurred in only 1.1% of cases.

The analysis of the types of breech presentation and maternal perineal damage of fetuses delivered vaginally is shown in Figures 7, 8. Complete breech presentations were the majority (61.2%) of all breech presentations delivered vaginally. Furthermore, among the 364 women we counted, 45.1% of cases had no perineal injury, 1.1% of cases had third degree or fourth degree lacerations, and 8.8% cases had lateral episiotomy.

**Figure 1:**
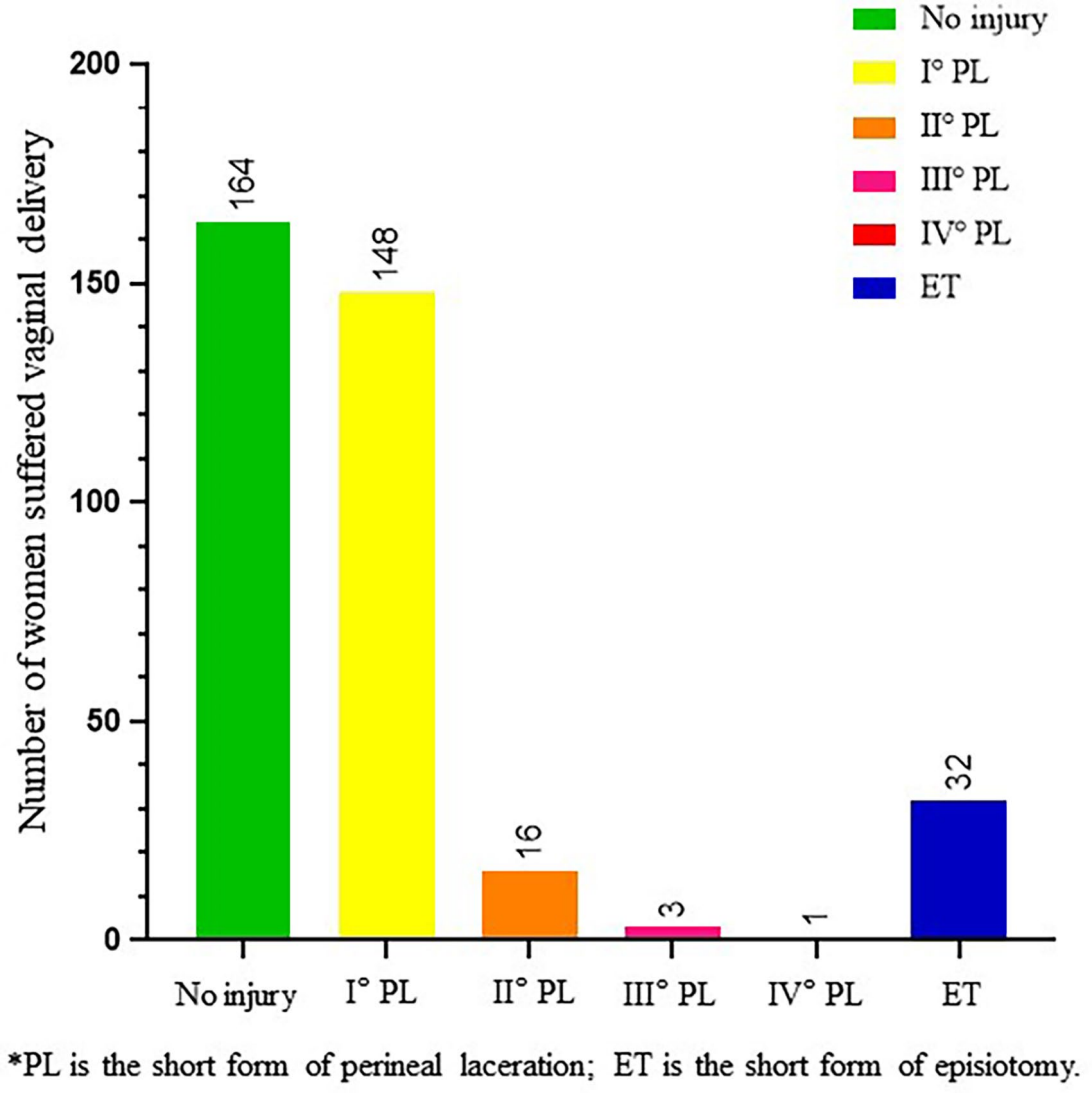
Study design. This figure shows the design of the overall study content. We divided 451 full-term singleton breech presentation parturient into planned vaginal delivery group (*n* = 429) and planned cesarean delivery group (*n* = 22), compared the obstetrical characteristics and neonatal outcomes between the two groups. And set a cephalic group, compared indicators mentioned above between cephalic group and breech one. We analyzed the indications for caesarean section in 17 emergency caesarean sections in the group planning for vaginal delivery, and compared the neonatal outcomes between it and the planned cesarean. Maternal perineal injury, stages of labor, types of breech presentation and 18 dead fetuses were analyzed in the vaginal delivery group.

Data on the second stage of labor is shown in Table 7. For primiparas, the proportion of prolongation of the second stage was 1.2%, while it was 1.6% for multiparas.

**Table 7:** Second stage of labor.

	Nulliparous	Multiparous
Mean time (min)	43.1 ± 33.2	35.0 ± 29.9
Prolonged	1 (1.2%)	5 (1.6%)

## 4. Discussion

### 4.1. Principal findings

Our study of deliveries on the Tibetan Plateau found that breech presentation fetuses planning a vaginal birth had a worse short-term prognosis than cephalic fetuses, but there was little difference in rates that converted to cesarean. For term breech presentation fetuses delivered in a typical lithotomy position, the short-term prognosis of CS appears to be better than that of vaginal breech delivery, but there appears to be no difference in short-term neonatal outcomes between elective CS and converting to CS after failed trial delivery. Furthermore, intrapartum death was the leading cause of perinatal death. Improving the operative skills of medical staff, improving medical conditions, timely recognizing dystocia or fetal distress during breech vaginal delivery and encouraging to convert to cesarean is expected to improve the safety of breech vaginal delivery.

### 4.2. Results

In our cohort of 451 singleton term breech fetuses, perinatal mortality rate in the planned vaginal delivery group with breech presentation was 4.2%, while the cephalic group was just 1.5% (*p* = 0.012). Intrapartum deaths accounted for 61.1% of total deaths in breech group. Mortality rates were higher than those reported worldwide by 0.3–1.3% (1, 6) in both cephalic and breech presentation, and the breech group was even higher. This was associated with the limited medical care in the Tibetan plateau area, the poor compliance of pregnant women, the irregularity of obstetric examinations, and the influence of religion and culture. In fact, in Tibet, China, many women lacked awareness of prenatal checkups. Although the government and national health organizations actively disseminated information about pregnancy and have introduced many policies to encourage regular maternity checkups in Tibet, a significant number of pregnant women refused regular prenatal check-ups and failed to detect breech presentation fetuses in time. Their refusal to undergo prenatal check-ups stemmed mainly from their religious beliefs. They believed in their God’s guidance and believed that children were God-given treasures, and whether children were healthy or not was God’s will and cannot be interfered with. The second reason was that Tibet was sparsely populated, the distance to the hospital was so long, that each prenatal check-up took a long time. And childbirth was a matter that local mothers go through many times, so they were not willing to waste time in this.

There were no deaths in either the planned CS group or the emergency CS group for breech vaginal delivery, although the number of CSs in this study was limited, which may have affected the accuracy of the conclusions to some extent, but it was undeniable that in Tibet, under the current medical and cultural background, CS had played a non-negligible role in ensuring the safety of breech delivery.

The number of infants with a low Apgar score at 1 min in the planned vaginal delivery group with breech presentation was significantly higher than that in the CS group, but there was no significant difference at 5 min. Apgar scores are widely used as diagnostic tests for asphyxia (22), and the diagnosis of neonatal asphyxia is based on the 1-min Apgar score. However, numerous studies have shown that delayed Apgar scores are more predictive of severe neonatal incidence than 1-min scores (23–25). A study suggested that full-term infants with an Apgar score below 7 at 5 min were associated with an increased risk of neonatal morbidity, mortality, and neurological deficits (26). Therefore, a low 1-min score may be of limited clinical importance to our study (27). In addition, previous studies show that the 1-min Apgar score in infants delivered vaginally is lower than that in a cesarean delivery group, while the difference in long-term prognosis was not obvious (27–29). This suggests that in our study, the long-term prognosis of newborns in the planned vaginal delivery group may not be worse than in the planned CS group. However, it should not be ignored that the low 1-min Apgar score may be owing to the lack of response to problems encountered during childbirth and neonatal rescue and resuscitation, which to some extent reflects a lack of skills of midwives and neonatologists and the limited medical conditions.

In our study, the incidence of serious neonatal complications was significantly higher in the breech group (11.7%) than in the cephalic group (1.9%) (*p* < 0.01), while the proportion of neonatal complications in the breech group was highest in neonatal encephalopathy. NE may result from acute or chronic hypoxic–ischemic injury, brain malformations, vascular injuries (including stroke), inborn errors of metabolism, and other causes. The diagnosis of Hypoxic–Ischemic Encephalopathy (HIE) was often popular in the United States and other high resource settings, which can be graded as mild, moderate and severe. Due to limited medical resources, we cannot clearly distinguish the etiology of NE in time, but HIE is the main cause of NE (Volpe’s study indicated that 50–80% NE cases were considered to have HIE, based on clinical, electroencephalographic (EEG), and MRI criteria) (30), and its prognosis may be different from other neonatal encephalopathies. For example infants with 5 min Apgar score > 7 and NE are unlikely to have long term adverse neurological outcomes due to birth asphyxia (17). Among the 18 deaths in the current study, hypoxic asphyxia was the chief cause, which indicated that hypoxic asphyxia was an important cause of poor neonatal prognosis for fetuses born in breech vaginal delivery in the Tibetan plateau. Breech presentation is an abnormal fetal orientation, and breech vaginal delivery is more likely to cause dystocia and hypoxia than cephalic delivery. However, our study found no significant difference in rates that converted to cesarean between the two groups, and that the breech group had even lower than the cephalic group (4.0% in the breech group versus 4.6% in the cephalic group). It has to be admitted that the lack of medical resources and personnel may be the main reason for this result. Compared to areas with abundant medical resources [29% in some European countries (31)], the rate in Tibet was too low. In fact, at Naqu People’s Hospital, every obstetrician was responsible for the delivery of at least 10 women. This was likely to result in a significant proportion of pregnant women with dystocia or fetal distress not being detected in time, resulting in adverse outcomes. This was also illustrated by the low Apgar score rate in the breech group (compared to the cephalic group). The incidence of low scores was significantly higher in the breech group than in the cephalic group, on both one-minute and five-minute scores. This suggested that breech vaginal delivery was a higher risk than in the cephalic delivery.

But is the option of planned vaginal breech not worth supporting? No. Some studies conducted in Finland have shown that neonates delivered vaginally in the breech presentation in high resource settings with hospital specific inclusion criteria and labor management protocols do not have a significant difference in prognosis compared with the head presentation (apart from Apgar suppression), nor does there be a clear difference in the experience of childbirth (32, 33). This suggests that the safety of breech vaginal delivery remains positive under conditions of rigorous selection and good medical care. In addition, CS has adverse effects on future pregnancies including a rate of uterine rupture in labor of approximately 0.5% in women with one prior cesarean delivery. Uterine rupture may be dramatic with the fetus extruded through the uterine opening requiring urgent cesarean with the potential for considerable maternal and fetal morbidity and mortality. Kenichiro’s study indicated that lower maternal educational level was an independent risk factor for uterine rupture among women with prior CS (34). In China, the maternal near miss and stillbirth rates in women with UR were, respectively, 2.35 and 2.12% (35). Due to limited medical resources in Tibet pregnant women often cannot receive timely treatment due to the long journey to seek medical treatment, however with the increase of time (more than 30 min), the adverse neonatal outcomes are increased (36). Ensuring the safety of mothers and babies giving birth in the breech presentation in Tibet requires improving the local medical environment and improving the professional level of medical personnel.

There is literature showing that increasing training for vaginal delivery in the breech presentation of a single fetus can improve the safety of vaginal delivery (37, 38). A study shows that while providing a short-term training program does not change the overall vaginal breech birth rate, it still makes sense for changes in clinical practice, such as the choice of different breech birth positions (39). In addition, a study has proposed that the all-fours position has a better safety profile than classical lithotomy delivery in breech delivery (40). Therefore, improving the professional skills of relevant medical personnel and the medical conditions they work in may be an important part of improving the safety of breach vaginal delivery in Tibet.

Manley’s study shows that effective neonatal resuscitation improves neonatal hypoxia and reduces disability in children who survive perinatal asphyxia (41). Oxygen levels in the Tibetan plateau are thinner than in the plains, so improving neonatal resuscitation and rescue techniques is particularly important. This may be an effective way to reduce maternal complications during pregnancy and even maternal mortality in the breech presentation at term during vaginal delivery.

In addition, we found no clear difference in short-term neonatal outcomes for converted CS compared to elective CS. This suggested that timely selection of CS when dystocia or the intrauterine hypoxia can improve the safety of breech vaginal delivery. Wouldn’t this be a better option than recommending elective CS for all breech presentation fetuses?

On the other hand, the special religious beliefs in Tibet were also a reason for the low CS rate. Some traditional ideas appear to diverge from modern medical knowledge. Therefore, through strengthening scientific education and popularizing modern medical knowledge in Tibet to balance the influence of traditional Tibetan beliefs with current scientific knowledge regarding birth outcomes is a direction that should be worked towards in the future. The ultimate goal is to promote the lives and health of mothers and children in Tibetan areas.

The average duration of the second stage of labor for both primipara and multipara in our study was longer than the data reported worldwide (40 min for primipara and 14 min for multipara) (42). McKinney JR’s study showed that the combined incidence of maternal and neonatal morbidity increases with the duration of the active phase and second stage of labor (43). For full-term fetuses, the second stage of labor with breech presentation is different from that in the cephalic presentation. When the second stage of labor is too long, it may indicate signs of cephalopelvic disproportion and require emergency CS (42). Therefore, obstetricians should pay more attention to the progress of labor, especially the second stage of labor, and appropriately ease the indications of CS to ensure the safety of mothers and infants.

In our study, the rate of third degree or fourth degree perineal laceration and lateral resection was lower than that reported abroad (44, 45). We think these may be associated with low neonatal weight in highland areas and multiple births in pregnant women. However, third degree or fourth degree perineal lacerations can still cause serious problems. It is beneficial to reduce the rate of severe perineal lacerations to improve the safety of vaginally delivered term breech fetuses. There is a study showing that hot compresses and massage can reduce third degree and fourth degree perineal lacerations, and not intervening in advance may reduce the rate of episiotomy (46). CNGOF guidelines recommend that perineal massage during pregnancy can reduce episiotomy rates and pain in the perineum and anus after childbirth. Furthermore, perineal massage or hot compresses during the second stage of labor can reduce the risk of anal sphincter injury (44).

It has to be mentioned that, owing to poor dependence, even though we had repeatedly emphasized the risk of vaginal breech delivery to patients, they still refused CS; this was also a major factor affecting the risk of maternal and infant outcomes in this study.

### 4.3. Clinical implications

Due to the high risk of breech presentation delivery, CS has become the optimal delivery method for breech presentation in the economically developed areas of China and even in most countries in the world. Studies on vaginal delivery, especially randomized controlled studies, have become increasingly rare. But the impact of CS on maternal injury and future pregnancies cannot be ignored (for example increased blood loss, infectious morbidity, longer hospitalization, abnormal placentation, risk for thromboembolic phenomenon, risk for uterine rupture and maternal mortality) (47). Due to the unique religious and cultural background and poor medical environment in Tibet, it is particularly important to ensure the health of mothers and children in breech presentation. And with the opening of the three-child policy in China, further research is needed to improve the safety of breech vaginal delivery and strive to provide safer and less damaging options for those pregnant women.

### 4.4. Research implications

Our study found that in the Tibetan Plateau region, the majority of pregnant women with breech presentation babies still chose vaginal delivery, although the short-term prognosis of CS may be better. However, if it is possible to improve the safety of vaginal delivery of women in special areas such as Tibet by improving medical conditions, improving the skill sets of relevant medical personnel, and timely recognizing dystocia or fetal distress during breech vaginal delivery and encouraging to convert to cesarean, perinatal mortality will be greatly reduced in these areas.

### 4.5. Strengths and limitations

The strength of this study lay in the fact that its large data base is convincing. And, the study was conducted in the highland area. However, we realize that there are still some limitations to our study. There were low CS rates in our data, which may have contributed to some bias. In addition, all women in our study were delivered in the lithotomy position, lacking relevant controls for delivery in other positions (like a semirecumbent or an all-fours position), it makes the conclusions relatively limited. Our research shows that in the future, we should focus on the training of obstetricians in Tibet on breech delivery techniques and neonatologists on neonatal resuscitation and rescue, strengthening the introduction of obstetricians in Tibet, and strive to improve the safety of breech vaginal delivery.

## 5. Conclusion

Our study shows that in the Tibetan Plateau region, for singleton term breech presentation fetuses delivered in the lithotomy position, vaginal delivery was less safe than for cephalic presentation fetuses. However, if dystocia or fetal distress can be identified in time and then encouraged to convert to cesarean, its safety will be greatly improved. On the Tibetan Plateau, a relatively large proportion of women choose vaginal delivery, so it is necessary to improve the safety of full-term breech vaginal delivery.

## Data availability statement

The original contributions presented in the study are included in the article/supplementary material, further inquiries can be directed to the corresponding author/s.

## Ethics statement

The studies involving human participants were reviewed and approved by the Ethics Committee of Naqu People’s Hospital. Ethics Number: 20200601. Written informed consent was not provided as this is a retrospective study.

## Author contributions

ZX developed the idea and design of this study. FL and DG were responsible for collecting, sorting, typing and analyzing data. KY wrote the manuscript. DZ, XX, and ZS critically reviewed the manuscript. All authors contributed to the article and approved the submitted version.

## Funding

This study was supported by the Tibet Local Science and Technology Project guided by the Central Government (grant no. XZ202001YD0005C), Scientifical Funds of Medical Assistance Program for Tibet from the Tibet Health Committee [grant nos. XZ2020ZR-ZY77(Z) and XZ2020ZRZY78(Z)], Natural Science Funds of Liaoning (grant no. 2019-BS-073), and Scientific technology project of Liaoning Education Administration (grant no. LZ2019044).

## Conflict of interest

The authors declare that the research was conducted in the absence of any commercial or financial relationships that could be construed as a potential conflict of interest.

## Publisher’s note

All claims expressed in this article are solely those of the authors and do not necessarily represent those of their affiliated organizations, or those of the publisher, the editors and the reviewers. Any product that may be evaluated in this article, or claim that may be made by its manufacturer, is not guaranteed or endorsed by the publisher.
